# Local Misalignment Scoring Reveals Spatially Uniform Chondrocyte Disorganization in a Wnt5a-C83S Knock-in Model of Robinow Syndrome

**DOI:** 10.21203/rs.3.rs-8283811/v1

**Published:** 2025-12-17

**Authors:** Zhendong A. Zhong, Megan N. Michalski, Cassandra R. Diegel, Cheryl Christie, Zachary Klamer, Nathan J. Lanning, Brian B. Haab, Stephanie Grainger, Bart O. Williams

**Affiliations:** Department of Cell Biology, Van Andel Institute, Grand Rapids, MI 49503, USA

**Keywords:** Wnt/PCP signaling, *Wnt5a*, Robinow Syndrome, chondrocyte orientation, limb development, local misalignment score (LMS), CRISPR/Cas9 mouse model, cell polarity

## Abstract

The WNT5A-mediated Wnt/Planar Cell Polarity (Wnt/PCP) pathway plays a key role in vertebrate development, particularly in limb morphogenesis. Robinow Syndrome (RS) is a rare genetic disorder characterized primarily by craniofacial malformations and limb shortening that is linked to mutations in multiple Wnt/PCP genes. The pathogenic *WNT5A* point mutation, Cys83Ser (C83S), is one of the most-studied RS-associated variants to date. It has been described as a loss-of-function, hypomorphic, or dominant-negative variant based on overexpression studies *in vitro* and *in vivo*. However, a mammalian model that mimics the C83S condition in human RS patients has not yet been established, and methods to distinguish between Wnt/PCP loss-of-function and gain-of-function *in vivo* phenotypes remain limited.

In this study, we present a novel image-based method, local misalignment score (LMS), for *in situ* visualization and quantification of cell alignment within long bones during late embryonic development, providing a reliable and specific *in vivo* readout of aberrant Wnt/PCP-associated phenotypes. Using this method to assess chondrocyte orientation across mouse limb regions, we found that the heterozygous germline *Wnt5a*-C83S point mutation in mice induces profound chondrocyte orientation defects. This phenotype is distinct from the disrupted chondrocyte orientation with spatially patterned severity observed in homozygous Wnt5a conditional knockout (*Wnt5a-*cKO) limbs, which represent a Wnt5a loss-of-function model, and from those in *Wnt5a-*LSL knock-in limbs, where ectopic Wnt5a expression disrupts the endogenous gradient rather than producing a true Wnt5a gain-of-function effect.

We further performed a comprehensive *in vitro* analysis of *Wnt5a*-C83S in C3H10T1/2 cells using a luciferase-based KIF26B reporter system along with other established Wnt signaling readouts. The results show that the C83S mutation does not exert dominant-negative effects on Wnt/PCP signaling, consistent with our *in vivo* findings.

In summary, our work provides new insights into the putative gain-of-function or neomorphic nature of the RS-related *WNT5A* mutation and its impact on WNT5A gradient-dependent limb development. We highlight the reliability of LMS as an *in vivo* morphological measure of chondrocyte orientation that reveals defects linked to aberrant Wnt/PCP activity. When combined with other spatially resolved readouts, LMS enables location-based evaluation of pathogenic mechanisms.

## Introduction

Robinow Syndrome (RS) is a rare inherited disorder characterized by developmental defects including craniofacial abnormalities and long bone shortening. RS can follow either autosomal dominant or autosomal recessive inheritance patterns, with the dominant form typically presenting milder phenotypes than the more severe recessive form [1]. The Wnt/Planar Cell Polarity (Wnt/PCP) signaling pathway is one of the multiple non-canonical (β-catenin-independent) Wnt signaling pathways, which includes the core PCP proteins VANGL1/2, PRICKLE, and DISHEVELLED [2]. The Wnt/PCP pathway plays a pivotal role in tissue and organ development by regulating key processes such as cell polarity, tissue morphogenesis, cell differentiation, and cell migration [3]. Most RS-associated genes identified to date are involved in the Wnt/PCP signaling pathway [4]. In limb development particularly, the Wnt/PCP pathway regulates the initiation of limb buds, digit patterning, joint formation, limb rotation, and the establishment of the proximal-distal axis [5–8]. Autosomal dominant mutations in Wnt5A frequently involve mutations in highly conserved residues that facilitate correct protein folding and function. One of the most extensively studied *WNT5A* pathogenic variants is the C83S substitution (c.248G>C; p.Cys83Ser). Analyses in zebrafish and Xenopus models have shown that WNT5A-C83S and the related WNT5A-C83R variants retain partial activity, consistent with a hypomorphic rather than null or dominant-negative classification [9]. In contrast, avian retroviral delivery of WNT5A-C83S into chicken embryonic limbs led to dominant-negative effects on chondrogenesis and limb and mandibular development [10, 11]. However, these findings are based on overexpression studies in non-mammalian models, and a physiologically relevant mammalian model carrying the C83S mutation has not been available.

Insights from mice globally deficient in Wnt5a show that only homozygous knockouts, not heterozygotes, exhibit PCP-related phenotypes in multiple organs [12, 13], suggesting that partial reduction of Wnt5a is generally well tolerated, at least with respect to RS-like skeletal features. In the mouse developing limb, Wnt5a is expressed in a well-described proximal-distal gradient with highest levels in the distal mesenchyme and apical ectodermal ridge, beginning as early as embryonic day (E)9.5 in mouse development [14]. Interestingly, both global loss (homozygous knockout) and ectopic overexpression of Wnt5a in mice can lead to comparable limb abnormalities [15], highlighting the importance of maintaining precise Wnt5a levels that sustain gradient regulation during limb development. We therefore reason that it is unlikely that the heterozygous C83S variant found in RS patients is a simple loss-of-function allele. Instead, C83S may exert its dominant effects through dominant-negative, gain-of-function, or neomorphic mechanisms that disrupt the spatial or temporal dynamics of Wnt5a signaling.

Most studies of Wnt/PCP signaling in the limb have been performed at E10.5–13.5 (early limb outgrowth and active mesenchymal condensation) in mice or Hamburger–Hamilton (HH) stages 18–28 (early elongation phases) in chicks [16, 17], because early limb buds contain relatively homogeneous mesenchymal cell populations (covered by surface ectoderm) that facilitate measurement of cellular morphology and behaviors. Current approaches capture the orientation of core PCP proteins (such as VANGL2) in mesenchymal progenitor cells, or the orientation of the cells themselves, as a readout for Wnt/PCP activity at these early developmental stages [5]. We asked whether similar measurements would be informative at later time points during limb development. This is particularly relevant because long bone shortening observed in RS and many other PCP-defective models becomes more apparent at later stages in development. More well-developed limbs contain a greater number and diversity of cell types that include differentiated chondrocytes, osteoblasts, and surrounding connective tissue, providing a biological context that more closely reflects the complexity of *in vivo* development. This richer context enables the evaluation of Wnt/PCP signaling across distinct cell populations and tissue interfaces, allowing for the detection of PCP defects and signaling outcomes that may not be apparent in more homogeneous early limb buds. Finally, orientation defects that originate during early morphogenetic processes, such as misaligned cell divisions or faulty cell intercalations, may accumulate over time, resulting in structural abnormalities that are more readily detectable at later stages. For example, mutations in zebrafish wnt11f1 and fzd7a/7b did not lead to phenotypes related with Wnt/PCP defects until 5 days post fertilization (dpf) [18].

Therefore, the goal of the current study was to determine whether cell orientation defects could be captured at later developmental stages (E14-E18, prior to birth) in mice as a readout for aberrant Wnt/PCP signaling underlying the RS-related *Wnt5a-*C83S pathogenic variant.

## Results

### 1. Quantitative alignment of chondrocytes provides a specific readout of defective Wnt5a/PCP activity in the limb

Wnt5a knockout in mice is a well-established model for defective Wnt/PCP signaling [2]. This model provides a foundation for assessing chondrocyte orientation as a morphological readout of Wnt/PCP disruption in more mature mouse embryonic limbs. To achieve limb-specific inactivation of Wnt5a, we generated a conditional knockout (*Wnt5a-*cKO) model using the limb mesenchyme-specific Prrx1-Cre driver [19]. Wild-type and *Wnt5a-*cKO embryonic limbs were collected at E18.5 analysis. As expected, *Wnt5a-*cKO mice had significantly shortened limbs (suppl_Fig.1).

**Fig.1:**
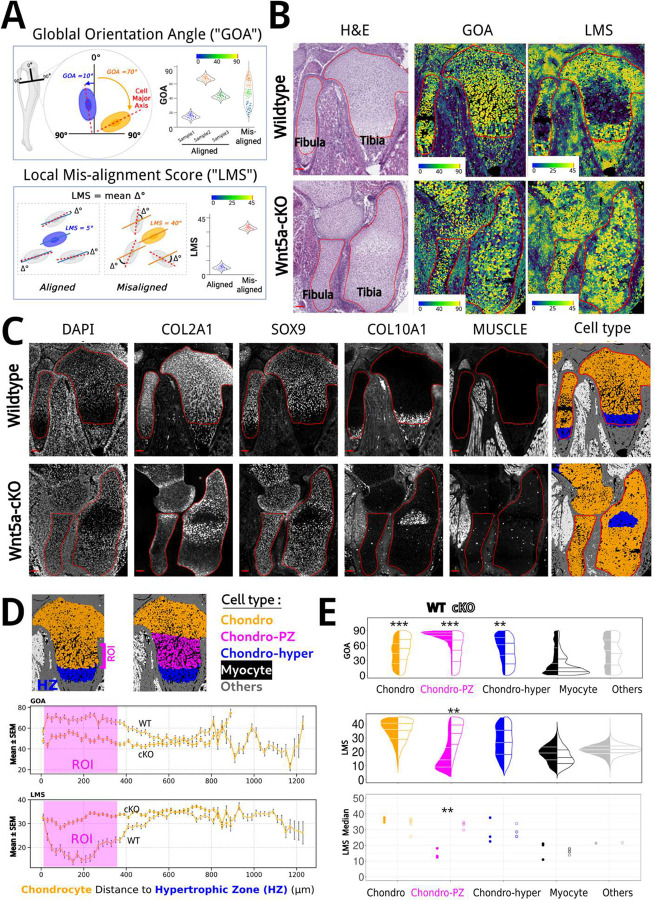
Chondrocyte Alignment Scoring GOA and LMS Highlight Wnt5a Loss-of-Function Effects A. Diagram of cell orientation measurements. GOA (Global Orientation Angle) quantifies each cell’s angular deviation from the proximal-distal tissue axis, while LMS (Local Misalignment Score) measures the consistency of local orientation within each cell’s neighborhood. Specifically, LMS is calculated as the average angular difference between a target cell and its neighboring cells within a 30 μm radius. Example violin plots illustrate distributions of GOA and LMS values for aligned versus randomly oriented cells. While LMS directly quantifies local orientation consistency, GOA does not measure alignment strength per se; instead, its population distribution reflects it, with narrow GOA distributions indicating strong alignment and broad distributions indicating weak alignment. B. H&E images and GOA and LMS heatmaps of E18.5 wild-type and *Wnt5a-*cKO hind limbs. The cartilage regions of the tibia and fibula are outlined in red. The GOA and LMS heatmaps display the distribution of cell orientation of whole limbs. Scale bars, 100 *μ*m. C. Immunofluorescence staining and cell type classification of wild-type and *Wnt5a-*cKO limb sections. Based on the staining shown, cells in the limb are segmented and classified as chondrocytes, hypertrophic chondrocytes, myocytes, and other cells, represented with pseudo colors. Note that COL10A1 shows non-specific staining in non-cartilage tissues but reliably marks hypertrophic chondrocytes within cartilage. The fluorescence intensity was adjusted to highlight specific staining and should not be compared across different sections. Scale bars, 100 *μ*m. D. Line plots showing spatial profiles of chondrocyte alignment metrics in wild-type and *Wnt5a-*cKO hind limbs. LMS and GOA values were calculated for chondrocytes (excluding the Chondro-hyper class) and grouped by their distance from the hypertrophic zone (HZ). The magenta shaded ROI marks the “chondro-PZ” population (cells adjacent to the HZ), which exhibit the most aligned orientation pattern in wild-type cartilage and the most pronounced difference in *Wnt5a-*cKO. N = 20079 chondrocytes for WT and N= 30360 chondrocytes for cKO. E. Violin plots showing the distribution of LMS and GOA values across cell types in wild-type and *Wnt5a-*cKO tibia and fibula. Filled violins represent wild-type, and open violins represent *Wnt5a-*cKO. The horizontal lines within each violin represent the 25th, 50th (median), and 75th percentiles. The plots highlight that chondro-PZ in *Wnt5a-*cKO limbs exhibit higher LMS values and broader GOA distributions compared to wild-type, indicating disrupted cell orientation. Chondro-hyper, myocyte, and other cell classes are included for comparison. Note that GOA for Others might not be accurate since the reference axis could not be reliably defined. The dot plot (bottom) summarizes median LMS values for individual embryos, with each dot representing one embryo. A permutation test based on the Wasserstein distance was used for GOA analysis, whereas LMS was analyzed using linear mixed-effects models with genotype × location interaction and a random embryo effect. Asterisks indicate significance: P < 0.05, P < 0.01, P < 0.001. N = 421 for chondro-hyper and N = 3000 to 25000 for other cell types from 3 to 5 embryos.

To quantify cell orientation we established two complementary angular measurements (Fig.1 A), namely the global orientation angle score (GOA) and the local misalignment score (LMS). GOA measures each cell’s alignment relative to the tissue axis (the angles represent cells’ deviation from the proximal-distal bone axis) and is conceptually similar to methods previously used to assess cell orientation in early limb buds and other developing tissues. LMS, by contrast, quantifies the mean angular difference between each cell and its neighboring cells (an arbitrary reference axis can be used for LMS, as the specific choice of axis is not critical). Importantly, LMS captures the consistency of cell orientations at the local level, providing an added layer of resolution to detect subtle disorganization. Comparing GOA and LMS-derived measurements of limb cell orientation, we observed significant differences in both GOA and LMS values between wildtype and *Wnt5a-*cKO bone elements (tibia and fibula), specifically in the cartilage regions (outlined in red) where Prrx1-Cre is active (Fig. 1B).

To quantify GOA and LMS in a cell-type-specific manner, we used cyclic immunofluorescence (CycIF) to stain cells in the limb for cell type classification [20]. We identified COL2A1, SOX9, COL10A1, and a polyclonal antibody (Muscle - 12/101) against newt skeletal muscle were suitable markers for chondrocytes, hypertrophic chondrocytes, and myocytes. DAPI and wheat germ agglutinin (WGA) were used to stain nuclei and cell membranes, respectively, which allowed us to segment cells using Cellpose and InstanSeg deep-learning-based software (suppl_Fig.2). We next classified chondrocytes, hypertrophic chondrocytes, and myocytes in both wild-type and *Wnt5a-*cKO limb samples collected at E18.5 (Fig.1 C).

The hypertrophic chondrocytes (COL2A1 and COL10A1 double positive) in the hypertrophic zone (HZ) are terminally differentiated cells at the end of the growth plate [21]. We examined the orientation of chondrocytes by plotting their orientation based on distance from the HZ. As shown in Fig.1 D, the chondrocytes adjacent to the HZ (0–400 *μ*m, highlighted in magenta) display the greatest difference in orientation (as measured with both GOA and LMS) between wild-type and *Wnt5a-*cKO limbs. In contrast, chondrocytes positioned farther from the HZ (> 400 *μ*m) were randomly oriented with less discernible difference between the genotypes. In addition, the distal end of the long bone (epiphysis) is spherical in shape, which makes angle measurements less accurate. We therefore focused our analysis on the middle region of the growth plate, which contains mostly proliferative zone (PZ) cartilage and allows for a more reliable assessment of chondrocyte orientation. We designated this target subgroup (highlighted in magenta in Fig.1D) as “chondro-PZ” while the remaining chondrocytes (excluding hypertrophic chondrocytes) are referred to as “chondro”.

As shown in Fig.1 E, among the classified cell types in wild-type limbs, chondrocytes (especially chondro-PZ) show an orientation mostly perpendicular (90° in GOA) to the proximal-distal axis, whereas myocytes adjacent to the bone tend to align more parallel with smaller GOA values. In contrast, chondrocytes in *Wnt5a-*cKO limbs exhibit disrupted orientation, reflected by a random distribution of GOA values and elevated LMS values. Among all cell types, the chondro-PZ population showed the most pronounced difference.

While GOA provides information about each cell’s orientation relative to the tissue axis, it does not indicate whether cells are aligned with each other, making it less informative and sometimes challenging to measure, especially in regions where the tissue axis is hard to define. In contrast, LMS measures the relative orientation among neighboring cells, which more directly reflects local organization and highlights subtle disorganization that may not be apparent with GOA. Therefore, we conclude that LMS is superior to GOA as an *in vivo* readout of disrupted cell alignment caused by defective Wnt/PCP activity in *Wnt5a-*cKO limbs.

To expand the applicability of LMS, we tested whether it could be performed on widely available H&E-stained sections. Cell segmentation and LMS measurements with H&E staining yielded similar LMS patterns along the proximal-distal axis as immunofluorescence-based measurements (suppl_Fig.3 A). However, H&E staining alone did not enable a distinction between chondrocytes and hypertrophic chondrocytes, limiting our ability to classify chondro-PZ cells. Therefore, LMS can also be applied to H&E-stained sections when chondro-PZ–specific quantification is not required or when these cells can be classified using other parameters such as cell size or cytoplasmic content. Beyond cartilage, H&E-based LMS measurement can be readily generalized to quantify cell orientation in other tissues where cell spatial organization is functionally relevant.

We next sought to determine whether LMS alterations are specific to Wnt/PCP signaling, rather than canonical Wnt signaling. While Wnt/PCP signaling regulates cell polarity, canonical Wnt signaling controls β-catenin stability and thereby influences limb development. We previously showed that mesenchyme-specific deletion of the canonical Wnt co-receptors Lrp5 and Lrp6 produced limb phenotypes closely resembling those of β-catenin–deficient embryos, which resulted in a loss of osteoblasts [22–24]. We determined the orientation of chondrocytes in Lrp5/6 double conditional knockout (*Lrp5/6-*cKO, which specifically ablates canonical Wnt signaling) and Wntless conditional knockout (*Wls-*cKO, which ablates all Wnt secretion and consequently suppresses both canonical and Wnt/PCP pathways) limbs [24]. In both models we used Col2a1-Cre, which enables gene knockout in virtually all limb chondrocytes [25]. As predicted, although *Lrp5/6-*cKO had significantly shortened limbs, the orientation of chondrocytes in the growth plate was not affected (suppl_Fig.3 B and C. In contrast, the shortened *Wls-*cKO limbs showed a random orientation of chondrocytes in the growth plate (suppl_Fig.3 B and D), similar to those observed in *Wnt5a-*cKO limbs. These results demonstrate that LMS specifically captures defects in Wnt/PCP signaling, rather than β-catenin–dependent canonical Wnt defects, making it a selective and robust readout of PCP–associated chondrocyte misalignment.

Furthermore, orientation defects in *Wls-*cKO limbs and normal orientation in *Lrp5/6-*cKO limbs were consistent across developmental stages from E14.5 to E18.5 (suppl_Fig.3B), indicating that LMS measurements of chondrocytes in the growth plate are not influenced by embryo age. This temporal robustness makes LMS well suited for analyzing later stages of limb development, where tissue complexity increases and traditional GOA-based methods become less reliable due to the difficulty in defining a consistent tissue axis. Moreover, LMS is particularly useful for tracking phenotypic progression in models where defects accumulate or change in severity over time.

Finally, we tested if the sectioning depth affects the LMS measurements in skeletal tissues. A wild-type E18.5 hind limb was serially sectioned longitudinally beginning at approximately the midpoint of the limb thickness, allowing us to capture both the optimal sectioning depth (middle layer of the growth plate) and less optimal regions (closer to the limb surface). We found that LMS measurements of chondrocytes are consistent when assessed at different anatomical layers of the limb, including both central and peripheral regions of the growth plate. (suppl_Fig.3 E).

In summary, we have established LMS as a reliable method to measure chondrocyte orientation in the growth plate of long bones, providing an *in vivo* readout for detecting Wnt/PCP-defective phenotypes that is independent of canonical Wnt signaling, developmental stage, or sectioning depth.

### 2. LMS measurements reveal spatially patterned limb defects in Wnt5a-modulated models

**Fig.2:**
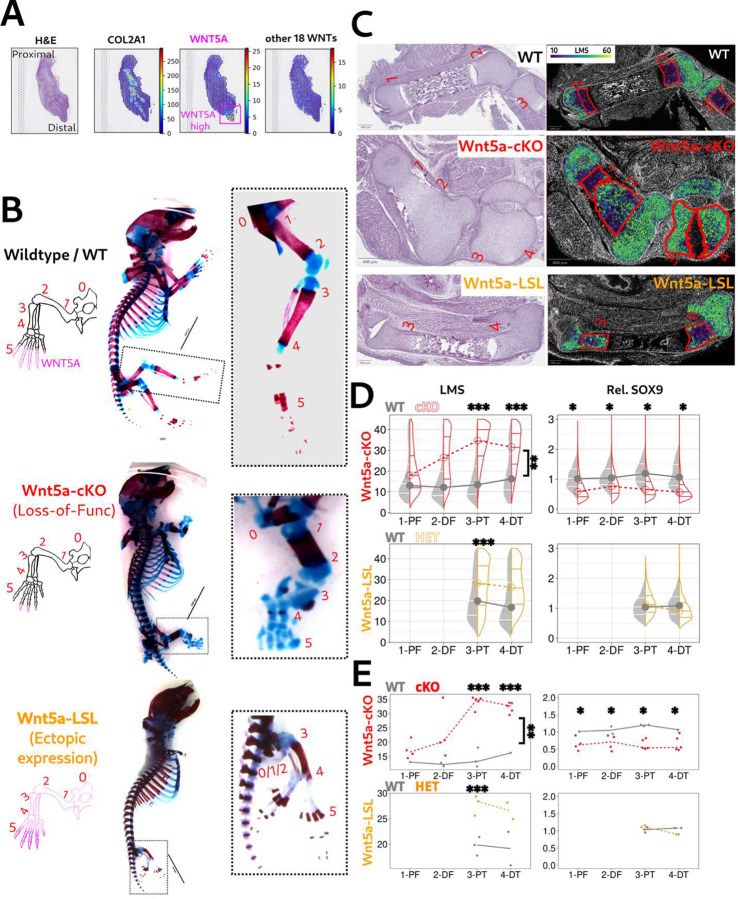
Spatial LMS Profiles Distinguish Phenotypes of Wnt5a Loss-of-Function and Ectopic Expression A. Spatial transcriptomics of human limb buds at PCW7 [26] reveals high *WNT5A* expression in the distal mesenchyme, which is like the Wnt5a expression pattern observed in mouse limb buds. Shown are the limb H&E image and the corresponding spatial transcriptomics data. The color scale represents gene expression levels for *COL2A1* (a chondrocyte marker indicating future skeletal elements), *WNT5A*, and the combined expression of all other 18 WNT genes (excluding *WNT5A*). Note that *WNT5A* displays significantly stronger expression in the distal limb bud, and its color scale differs from those used for other WNT ligands. The spatial transcriptomics data are from the E-MTAB-10367 dataset. B. Diagrams (with *WNT5A* expression shown in magenta) and whole-mount skeletal staining of E18.5 embryos from wild-type, limb-specific Wnt5a knockout (*Wnt5a-*cKO), and ectopic Wnt5a expression (*Wnt5a-*LSL) models. Zoom-in views of hind limbs with labeled skeletal elements (0-hip, 1-proximal femur, 2-distal femur, 3-proximal tibia, 4-distal tibia, 5-paws) highlighting phenotypic differences. In the *Wnt5a-*cKO diagram, residual Wnt5a expression is retained at the tips, reflecting the lack of Prrx1-Cre activity in the AER. In the skeletal staining, red (alizarin red) indicates mineralization of the bone elements, while blue (alcian blue) indicates cartilage. Scale bars, 5000 *μ*m. C. H&E staining and LMS heatmaps of hind limbs from wild-type, *Wnt5a-*cKO, and *Wnt5a-*LSL E18.5 embryos. The heatmaps (overlaid on DAPI fluorescence) display the distribution of LMS values across bone elements (chondrocyte only). The chondrocyte regions (including portions of the hypertrophic zones) in bone elements 1–4 are outlined in red. Note that not all four bone elements are shown for the wild-type sample due to longer bones, and *Wnt5a-*LSL limbs contain minimal cartilage in elements 0, 1 and 2. Scale bars, 400 *μ*m. D. Violin plots display LMS and relative SOX9 expression in chondro-PZ cells from *Wnt5a-*cKO (top) and *Wnt5a-*LSL (bottom) E18.5 hind limbs, grouped by bone element. SOX9 expression is normalized to the mean expression of wild-type chondro-PZ cells in element 1 for the *Wnt5a-*cKO model or element 3 for the *Wnt5a-*LSL model. Median LMS values are connected by lines to illustrate trends across bone elements. Statistical analyses were performed using linear mixed-effects models with genotype × location interaction. Importantly, LMS showed a significant genotype–location interaction (p = 0.01) in *Wnt5a-*cKO limbs but not *Wnt5a-*LSL limbs (p=0.58). Asterisks above each violin pair denote significant pairwise differences between wild-type and *Wnt5a-*cKO or *Wnt5a-*LSL limbs (* p < 0.05; ** p < 0.01; *** p < 0.001). Bone elements correspond to panel B. N = 5000 to 40000 chondro-PZ from 2 to 5 embryos per genotype. E. Line plots display embryo-level LMS (left) and relative SOX9 expression (right) across bone elements in wild-type (black/gray), *Wnt5a-*cKO (red), and *Wnt5a-*LSL (orange) E18.5 hind limbs. Each point represents the median value per embryo, and dashed lines indicate mean trends within each genotype. Statistical analyses were performed using linear mixed-effects models with genotype × location interaction as described in panel D. Asterisks denote significant pairwise differences between wild-type and mutant limbs (* p < 0.05; ** p < 0.01; *** p < 0.001). N = 2 to 5 embryos per genotype.

Wnt5a is highly expressed in developing mouse limbs and is the only Wnt ligand, among the 19 in the mammalian genome, that has been linked to Robinow Syndrome (RS) [12, 14]. Supporting this, spatial transcriptomics data from human limb buds at the 7th post-conception week (PCW7, dataset E-MTAB-10367) show high *WNT5A* expression in the distal limb mesenchyme and apical ectodermal ridge (AER) (Fig.2 A) [26]. While other Wnt ligands are also present in the human limb bud, their expression is markedly lower and does not spatially overlap with *WNT5A*. Based on these observations, we hypothesize that WNT5A functions as the primary Wnt/PCP signal during human limb development, with its gradient modulating pathway activity within the limb bud, as observed in mice.

We generated another *Wnt5a-*LSL mouse models in which a lox-stop-lox (LSL) cassette was inserted between the *Wnt5a* cDNA sequence and the ROSA26 locus [27]. When crossed with Prrx1-Cre, Wnt5a is ectopically expressed specifically in the limbs (Fig.2B bottom, and suppl_Fig.1). In parallel, the Prrx1-Cre-driven *Wnt5a-*cKO model was used as a control for the *Wnt5a-*mediated Wnt/PCP loss-of-function phenotype. As shown in Fig.2B, both *Wnt5a-*LSL and *Wnt5a-*cKO models show significant limb shortening, but the *Wnt5a-*LSL limbs lack the proximal limb elements (hip/femur of hind limb and scapula/humerus of forelimb), while the *Wnt5a-*cKO limbs show much more severe phenotypes within distal elements, with the most severe phenotypes occurring in the digits. These observations suggest that both elimination of, or ectopic expression of Wnt5a disrupts limb development in a Wnt5a gradient-dependent manner and highlight the importance of maintaining the spatial distribution of Wnt5a signaling for proper patterning along the proximal–distal axis.

To examine differences in phenotypic severity across skeletal elements in *Wnt5a-*modulated limbs, we first evaluated LMS in six bone elements (0–5): 0-hip, 1-proximal femur, 2-distal femur, 3-proximal tibia, 4-distal tibia, and 5-paws (Fig.2B). The hip and paw regions were subsequently excluded from analysis due to their anatomical complexity. As shown in Fig.2C and 2D, LMS heatmaps of E18.5 hind limbs from wild-type, *Wnt5a-*cKO, and *Wnt5a-*LSL embryos reveal that chondrocytes in *Wnt5a-*cKO limbs exhibit more pronounced orientation defects, reflected by elevated LMS values, particularly in the distal elements.

As a master regulator of chondrogenesis, the transcription factor SOX9 is essential for mesenchymal condensation and cartilage formation [28, 29]. Previous studies have shown that SOX9 expression is reduced in Wnt5a global knockout embryonic limb buds [15, 30], underscoring its value as a marker of *Wnt5a-*dependent chondrogenesis. Consistent with this, *Wnt5a-*cKO limbs displayed a significant reduction in nuclear SOX9 expression in chondro-PZ cells across all four bone elements (Fig.2D, top). In contrast, SOX9 levels in *Wnt5a-*LSL chondro-PZ remained largely unchanged (Fig.2D, bottom), indicating that ectopic Wnt5a expression perturbs chondrocyte orientation without affecting SOX9-driven chondrogenic identity.

Taken together, these spatially distinct phenotypes are consistent with a physiological Wnt5a gradient that is highest in distal regions of the limb bud. In the *Wnt5a-*cKO model, loss of Wnt5a in distal domains results in severe distal defects, while in the *Wnt5a-*LSL model, broad ectopic expression disrupts the native gradient and shifts the severity proximally. In conclusion, our findings support that physiological Wnt5a levels serve as a critical determinant of limb morphogenesis responding to Wnt5a modulation through a gradient-dependent mechanism. Alterations in Wnt5a expression, either a reduction or an elevation, result in limb malformations with spatially distinct severity patterns that reflect the degree of deviation from the physiological Wnt5a gradient. The *Wnt5a-*cKO model exemplifies a classical loss-of-function phenotype, while the *Wnt5a-*LSL model highlights the consequences of disrupting the gradient via ectopic Wnt5a expression. Unlike these models, we expect that a true gain-of-function Wnt5a model would exhibit increased Wnt5a expression or signaling activity while preserving the endogenous spatial gradient of Wnt5a within the limb bud. Such a model would enhance Wnt5a pathway output without altering the directionality or distribution of Wnt5a, allowing the gradient-dependent patterning mechanisms to remain intact (suppl_Fig.1E).

With this framework, we next asked whether the C83S, a heterozygous point mutation in *WNT5A* identified in human RS patients, would phenocopy a loss-of-function model like *Wnt5a-*cKO, as would be expected if C83S acted as a dominant-negative allele.

### 3. C83S mutation disrupts chondrocyte orientation distinct from cKO and LSL models

**Fig.3:**
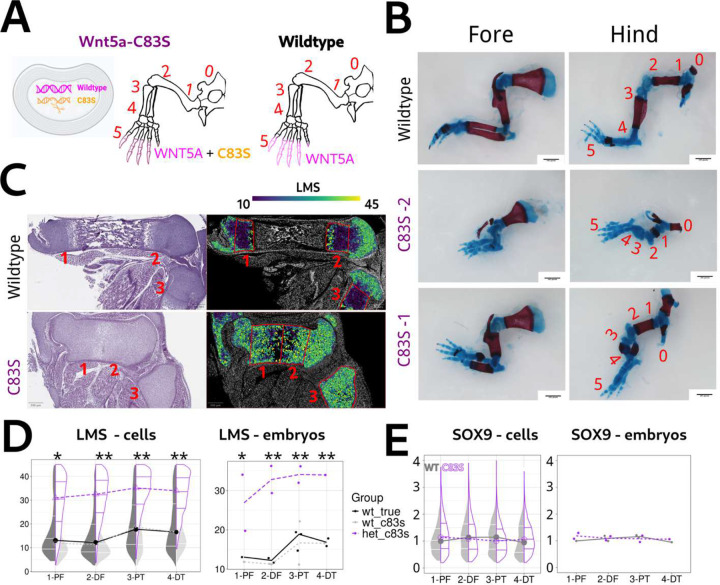
The Wnt5a-C83S mutation induces limb chondrocyte alignment defects distinct from Wnt5a knockout or ectopic expression models A. Diagram of the *Wnt5a-*C83S mouse model, in which the Cys83Ser point mutation was introduced into one of the two Wnt5a alleles using the CRISPR/Cas9 system. The hind-limb diagram shows the expression of Wnt5a gene in the limb bud, with wild-type Wnt5a in magenta and *Wnt5a-*C83S in orange. Note that the gradient of wild-type or *WNT5A*-C83S in the *Wnt5a-*C83S limb bud is not disturbed, as indicated by the unaltered color intensity across bone elements in the limb diagram. B. Whole-mount skeletal staining of E18.5 *Wnt5a-*C83S forelimbs and hind limbs, showing two CRISPR-edited embryos that represent the range of severity observed among the mutants. The red (alizarin red) indicates mineralization of the bone elements, while the blue (alcian blue) indicates cartilage. Scale bars, 1000 *μ*m. C. H&E staining and LMS heatmaps of E18.5 wild-type and *Wnt5a-*C83S hind limbs. The heatmaps are overlaid on DAPI fluorescence and display LMS values for all chondrocytes across bone elements 1, 2, 3, and 4, corresponding to the same anatomical regions shown in panels A and B. The cartilage regions, including both chondro-PZ cells and hypertrophic chondrocytes, are outlined in red within each element. Scale bars, 250 *μ*m. D. Violin plots and line plots display LMS values in chondro-PZ cells (left) and embryo-level medians (right) from wild-type (wt_c83s), *Wnt5a-*C83S heterozygous (het_c83s), and pooled wild-type (wt_true) E18.5 hind limbs from non-CRISPR-edited embryos, grouped by bone element. Median LMS values are connected by dotted lines to illustrate trends across elements. Statistical analyses were performed using linear mixed-effects models with genotype × location interaction as described in Fig.2 E. Asterisks indicate significant differences between wt_true and het_c83s (* p < 0.05). Bone elements correspond to panel B: 1, proximal femur (PF); 2, distal femur (DF); 3, proximal tibia (PT); 4, distal tibia (DT). N = 1400 to 10000 chondro-PZ cells for each bone element from 2 to 5 embryos. E. Violin plots and line plots showing relative SOX9 expression in chondro-PZ cells (left) and embryo-level medians (right) from wt_c83s and het_c83s E18.5 hind limbs, grouped by bone element. SOX9 expression is normalized to the mean of wt_C83S chondro-PZ cells in element 1. Median values are connected by dotted lines. No significant differences in SOX9 expression were detected between genotypes. Statistical analyses were performed as the panel D. N = 500 to 3000 chondro-PZ cells for each bone element from 2 embryos each genotype.

We next generated a mouse model carrying the Cys83Ser (C83S) point mutation in the Wnt5a gene using CRISPR/Cas9 to investigate how it influences mouse limb development. This model allows us to assess C83S effect on Wnt/PCP pathway function indirectly through surrogate readouts such as altered chondrocyte orientation. The C83S mutation was introduced into one of the two Wnt5a alleles, so cells were expected to carry both wild-type and C83S Wnt5a alleles (Fig3.A), which mimics the heterozygous state of human RS patients. Indeed, the heterozygous C83S mutation in Wnt5a leads to a significant shortening of the limbs (Fig.3 B) and face (suppl_Fig.4) in E18.5 embryos. The whole-mount skeletal staining of multiple E18.5 C83S limbs shows that the limb shortening is significant among the CRISPR-edited embryos with varying severity (Fig.3 B). Interestingly, the severity of the limb shortening appears much less correlated with the Wnt5a expression gradient in the limb bud, which is different from the *Wnt5a-*cKO and *Wnt5a-*LSL models (Fig.2B). In particular, C83S digits are among the least affected, aside from reduced mineralization, which stands in sharp contrast to *Wnt5a-*cKO limbs where the digits exhibit the most severe defects.

To investigate how the *Wnt5a-*C83S mutation alters chondrocyte orientation across limb elements, we performed LMS measurements on sections from wild-type and *Wnt5a-*C83S hind limbs. To control for potential off-target effects introduced during CRISPR/Cas9 editing, we also included wildtype embryos (“wt-true”) from *Wnt5a-*cKO and *Wnt5a-*LSL litters as additional controls. Because LMS is a cell morphology–based metric, it is less susceptible to batch effects in comparative analyses. In contrast to the *Wnt5a-*cKO model, chondro-PZ cells in *Wnt5a-*C83S limbs exhibit a consistent disruption of orientation (indicated by LMS) across the four limb elements (Fig.3 C and D). In addition, C83S chondro-PZ cells do not show reduced SOX9 expression compared to wild-type cells (Fig.3 E). These results suggest that the C83S mutation does not result in a simple loss-of-function phenotype like *Wnt5a-*cKO, nor does it mimic the ectopic Wnt5a expression phenotype observed in *Wnt5a-*LSL. Instead, it appears to act through a distinct non-loss-of-function mechanism that causes more uniform limb defects. This mechanism could involve either a gain-of-function or a neomorphic (gain of new function) effect, but it is not simply due to the loss of Wnt5a function from one allele.

### 4. *In vitro* characterization of C83S reveals partial loss-of-function without dominant-negative effects

To further investigate the functional consequences of the C83S mutation, we performed a series of *in vitro* experiments to examine the functional properties of the WNT5A-C83S protein and its interactions with Wnt/PCP signaling components.

**Fig.4:**
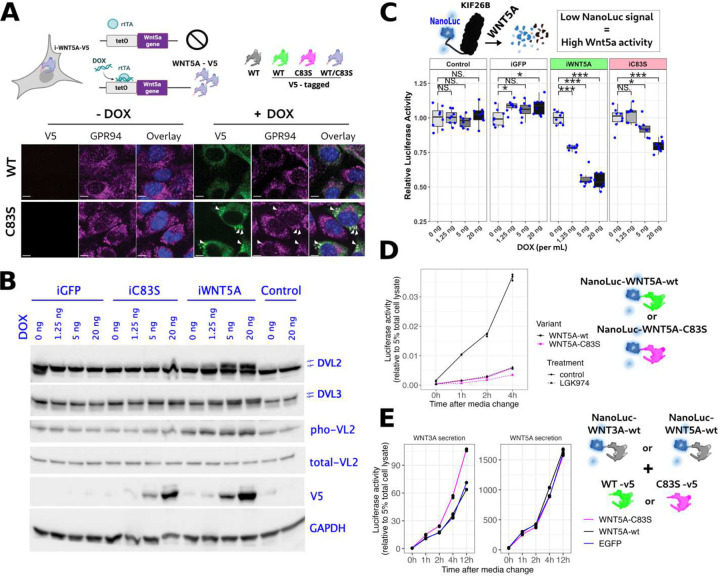
Overexpressed Wnt5a-C83S variant exhibits much reduced Wnt5a activity *in vitro* A. Diagram illustrates C3H10 cells stably engineered with a DOX-inducible WNT5A (V5-tagged) expression cassette. The colors of WNT5A proteins in the diagram indicate wild-type (untagged), V5-tagged WNT5A-WT, or V5-tagged C83S, with purple denoting either WNT5A-WT-V5 or WNT5A-C83S-V5. Immunofluorescence with anti-V5 and anti-GPR94 antibodies shows that both WNT5A-WT and WNT5A-C83S localize across the cytoplasm and endoplasmic reticulum. In addition, WNT5A-C83S accumulates in large GRP94-positive puncta (triangles), whereas WNT5A-WT appears more evenly distributed. Scale bars, 20 μm. B. Western blot analysis shows that WNT5A-C83S fails to induce phosphorylation of DVL2, DVL3, or VANGL2, in contrast to WNT5A-WT. Equal numbers of C3H10 cells stably expressing EGFP, WNT5A-WT, WNT5A-C83S, or parental cells (Control) were treated with doxycycline at the indicated concentrations for 16 hours. Blots shown are from multiple PVDF membranes loaded with the same samples and probed with the antibodies against phosphorylated VANGL2 and total DVL2, DVL3, VANGL2, V5, and GAPDH. C. KIF26B-Nanoluc reporter assay shows that WNT5A-C83S reduces reporter activity significantly less than wild-type WNT5A. The reporter, a luciferase-KIF26B fusion (KIF26B-NanoLuc), is degraded upon Wnt5a pathway activation, such that lower luciferase activity indicates stronger signaling. C3H10 cells expressing both the KIF26B-NanoLuc reporter and one of the indicated WNT5A variants were treated with increasing doses of DOX (0–20 ng/mL). Negative controls included KIF26B-NanoLuc–only cells (Control) and cells with inducible EGFP (iGFP). Statistical significance was assessed using Wilcoxon rank-sum test comparing each DOX-treated group to the corresponding non-DOX (0 ng) control. Asterisks denote significance levels: * p < 0.05; ** p < 0.01; *** p < 0.001; NS, not significant. D. WNT5A secretion assay with or without LGK974. NanoLuc was fused to the N-terminus of wild-type or C83S WNT5A, and luciferase activity in the culture media was measured and normalized luciferase activity in total cell lysate of the same cells at the indicated time points after media replacement in 293T cells stably expressing these constructs. LGK974 was used as a control to inhibit the secretion of all WNT ligands. E. Secretion assay measuring wild-type WNT3A or WNT5A secretion in the presence of co-expressed wild-type or C83S WNT5A. The NanoLuc–tagged WNT3A or WNT5A was stably expressed in HEK293T cells, which were transfected with either wild-type or C83S WNT5A for 48 hours. Luciferase activity in the media was measured at the indicated time points after media replacement to assess the secretion rate of the stably expressed wildtype WNT3A or WNT5A proteins to the culture media.

First, using the mouse embryonic mesenchymal cell line C3H10T1/2 (C3H10), we established stable lines with doxycycline (DOX)-inducible V5-tagged Wnt5a (Fig.4A, top), which showed low background activity and minimal growth inhibition in the absence of DOX, and upon induction produced high *Wnt5a* expression and robust signaling output. As shown in Fig. 4A (bottom), both wild-type WNT5A-V5 and WNT5A(C83S)-V5 proteins were broadly cytoplasmic and partially co-localized with the endoplasmic reticulum marker GRP94. Notably, WNT5A-C83S-V5 also formed large puncta that co-localized with GRP94 (an endoplasmic reticulum marker), indicating potential protein misfolding or aggregation. While such behavior could indicate impaired secretion or dominant-negative interference, additional functional assays are required to determine whether C83S interferes with wild-type WNT5A signaling. Phosphorylation of VANGL2 and DVL2/3 is a well-established downstream event of non-canonical Wnt/PCP signaling and serves as a biochemical indicator of pathway activation. Western blot analysis (Fig. 4B) showed that only wild-type WNT5A, but not WNT5A -C83S, induced phosphorylation of DVL2, DVL3, and VANGL2 in a DOX dose-dependent manner, suggesting C83S functions as a loss-of-function variant when overexpressed alone.

Second, we employed a reporter system based on degradation of KIF26B in response to non-canonical Wnt5a signaling as a primary readout of pathway activation (Fig.4C, top). KIF26B, identified by mass spectrometry as a downstream target of Wnt5a/Ror signaling [31], undergoes proteasomal degradation upon pathway activation. We fused the C-terminal region of KIF26B, which is responsive to WNT5A stimulation [32], to NanoLuc Luciferase (NanoLuc) and demonstrated that KIF26B-NanoLuc activity serves as a reliable readout of Wnt5a signaling. Using this assay (Fig.4C, bottom), we observed that DOX-induced expression of wild-type WNT5A caused a marked reduction in luciferase activity, whereas the C83S variant produced only a modest decrease even at high expression levels, consistent with a hypomorphic effect.

Finally, WNT5A ligand is secreted from producing cells and binds to its cognate receptors on neighboring cells or the same cells. Therefore, we asked if the C83S variant interferes with wild-type WNT5A secretion, thereby exerting a dominant-negative effect on Wnt/PCP signaling. To test this hypothesis, we first performed a WNT5A secretion assay (Fig. 4D) by fusing NanoLuc to the N-terminus of wild-type or C83S WNT5A and measuring luciferase activity in the culture media at various time points after media replacement. The results showed that secretion of the C83S variant into the media was significantly reduced compared with wild-type WNT5A. LGK974, a Porcupine inhibitor, blocked the secretion of both wild-type and C83S WNT5A. To further examine whether the C83S variant interferes with the secretion of wild-type WNT5A or other Wnt ligands (WNT3A is used as an example), we co-expressed luciferase-conjugated wild-type WNT3A or WNT5A with either wild-type or C83S WNT5A. The results showed that secretion of neither WNT3A nor WNT5A was affected by the presence of the C83S variant (Fig. 4E).

Since RS-associated *WNT5A* variants appear to act dominantly in human patients, and WNT5A-C83S does not impair wild-type Wnt ligand secretion (Fig.4E), we hypothesize that C83S may exert a dominant-negative effect on wild-type Wnt5a signaling by interfering with its function via mechanisms other than secretion. Given that WNT proteins are generally poorly secreted and can function as juxtracrine signaling molecules acting at the cell surface or within local microenvironments [33], we reasoned that co-expression of mutant and wild-type WNT5A within the same cellular context could reveal potential competitive or interference effects. To test this hypothesis, we designed several additional cell culture models in which both wild-type and C83S WNT5A were examined within the same system.

**Fig.5:**
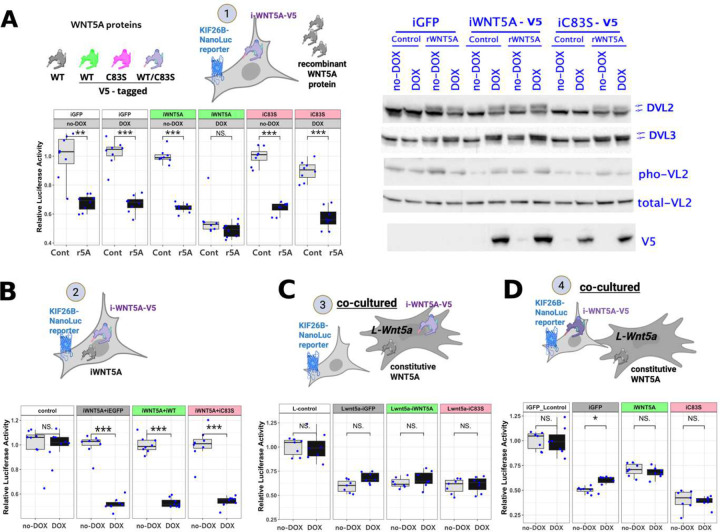
The Wnt5a-C83S variant does not exert dominant-negative effects on wild-type Wnt5a signaling A. Model 1. WNT5A-C83S expression in recipient cells does not block recombinant WNT5A (r5A)-induced signaling. C3H10-KIF26B/NanoLuc cells stably expressing inducible V5-tagged WNT5A-WT or C83S were treated with recombinant WNT5A (100 ng/mL) for 16 hours, with or without doxycycline co-treatment before luciferase measurement. Blots shown are from multiple PVDF membranes loaded with the same samples and probed with the antibodies to detect phosphorylation of DVL2, DVL3, and VANGL2. Note that cells with induced wild-type WNT5A-V5 expression did not exhibit additional response to recombinant WNT5A, consistent with saturated signaling output from DOX-induced WNT5A-V5. The colors of WNT5A proteins in the diagram indicate wild-type (untagged), V5-tagged WNT5A-WT, or V5-tagged C83S, with purple denoting either WNT5A-WT-V5 or WNT5A-C83S-V5. B. Model 2. WNT5A-C83S expression does not inhibit co-expressed wild-type WNT5A signaling in the same cells. Equal numbers of C3H10- KIF26B/NanoLuc cells stably expressing inducible WNT5A (untagged) and V5-tagged WNT5A (Wildtype or C83S) were treated with DOX for 16 hours before luciferase activity was measured. Parental C3H10-KIF26B/NanoLuc (control) cells (no exogenous WNT5A expression) served as a negative control; C3H10-KIF26B/NanoLuc cells stably expressing inducible WNT5A (untagged) with inducible EGFP expression (iWNT5A+iEGFP) served as a positive control. C. Model 3. WNT5A-C83S expression in donor cells does not inhibit wild-type WNT5A functioning from the donor cells. C3H10-KIF26B/NanoLuc reporter cells were co-cultured at a 1:1 ratio with L-Wnt5a cells stably expressing untagged WNT5A (constitutively expressed) and V5-tagged WNT5A (WT or C83S, inducible). Donor L-Wnt5a cells were pre-treated with/without doxycycline (DOX) for 24 hours prior to co-culture, which continued for an additional 16 hours before luciferase activity was measured. L parental cells (L-control, with no exogenous WNT5A expression) served as a negative control, and inducible EGFP-expressing L-Wnt5a cells (Lwnt5a-iGFP) served as a positive control. D. Model 4. WNT5A-C83S expression in recipient cells does not interfere with responding to wild-type WNT5A from donor cells. C3H10-KIF26B/NanoLuc cells stably expressing V5-tagged WNT5A-WT or C83S were co-cultured at 1:1 ratio with L-Wnt5a cells (with or without doxycycline treatment) for 16 hours before luciferase activity was measured. Reporter cells expressing WNT5A-C83S did not block wild-type WNT5A from L-Wnt5a donor cells from activating the reporter.

- Model 1 (paracrine, Fig. 5A). Either wild-type or C83S is inducibly expressed in C3H10-KIF26B/NanoLuc cells, and recombinant WNT5A protein (r5A) is added to the culture media. This model tests whether WNT5A-C83S can inhibit WNT5A-WT signaling in a paracrine (secreted factor) manner. If C83S was dominant-negative, the DOX-induced C83S expression would be expected to block the signaling output from recombinant WNT5A protein.
- Model 2 (autocrine, Fig. 5B). WNT5A-WT (inducible and untagged) and WNT5A variant (inducible and V5-tagged, wild-type or C83S) are co-expressed in the same C3H10-KIF26B/NanoLuc cells. This model tests whether WNT5A-C83S can inhibit the untagged WNT5A-WT signaling in an autocrine manner. If C83S was dominant-negative, the induced C83S expression would be expected to block the signaling output from the induced/untagged WNT5A-WT from the same cells.
- Model 3 (juxtracrine, Fig. 5C). V5-tagged WNT5A (either wild-type or C83S) is co-expressed in the “L-Wnt5a” cells, in which untagged and wild-type WNT5A is stably and constitutively expressed. These L-Wnt5a cells are then co-cultured with C3H10-KIF26B/NanoLuc reporter cells. This model tests whether WNT5A-C83S can inhibit WNT5A-WT signaling in a juxtracrine (cell-to-cell) manner. For example, if WNT5A-C83S was dominant-negative and impaired membrane retention or secretion of WNT5A-WT from L-Wnt5a cells, the reporter/recipient cells should show reduced response to WNT5A-WT (untagged) from L-WNT5A cells co-expressing WNT5A-C83S.
- Model 4 (juxtracrine, Fig. 5D). Similar to Model 1, but instead of recombinant WNT5A, wild-type WNT5A is provided by co-culturing with L-Wnt5a cells that stably express wild-type WNT5A. If C83S was dominant-negative, the DOX-induced C83S in the reporter cells would be expected to block the signaling response to wild-type WNT5A from the L-Wnt5a cells.

These cell culture models allowed us to comprehensively test whether C83S acts in a dominant-negative manner on wild-type Wnt5a signaling in various cellular contexts. Across all four models, induction of C83S expression did not reduce the signaling output from wild-type WNT5A, whether supplied as recombinant protein, co-expressed in the same cells, presented on neighboring cells, or provided by co-cultured donor cells. In each case, reporter activity in the presence of C83S was comparable to that of control conditions with wild-type WNT5A alone or co-expressed with EGFP, indicating that C83S does not inhibit wild-type WNT5A from functioning.

In summary, our *in vitro* results suggest that the WNT5A-C83S variant does not exhibit a dominant negative effect on wild-type WNT5A from signaling or secretion in any of the tested cellular contexts. We conclude that the C83S mutation may not interfere with wild-type WNT5A’s ability to activate Wnt/PCP signaling based on currently available *in vitro* readouts, which is consistent with the limb defects observed in our C83S mouse model, phenotypically distinct from the *Wnt5a-*cKO loss-of-function model.

## Discussion

In this study, we established the Local Misalignment Score (LMS) as a novel *in vivo* readout to compare chondrocyte orientation across wild-type, *Wnt5a-*cKO, *Wnt5a-*LSL, and *Wnt5a-*C83S mice. Wild-type limbs showed organized chondrocyte alignment, whereas *Wnt5a-*cKO and *Wnt5a-*LSL models exhibited disrupted orientation with spatially patterned severity across limb elements. In contrast, C83S mutants displayed uniform orientation defects and distinct limb abnormalities, indicating that the variant may act through a gain-of-function or neomorphic mechanism, a conclusion reinforced by our *in vitro* assays.

### Strengths and limitations of LMS

Unlike previous approaches for analyzing limb cell orientation (including GOA in current study), which measure deviation from the tissue axis, LMS quantifies local alignment among neighboring cells. This local approach makes LMS more robust and easier to visualize on tissue sections, particularly in regions where the tissue axis is difficult to define. During late stages of limb development, when chondrocytes rapidly proliferate, elongate, and align in a Wnt/PCP-dependent manner, LMS effectively detects changes in cellular organization associated with disrupted Wnt/PCP signaling. Importantly, LMS measurements are not influenced by canonical Wnt inhibition, embryo age, or tissue sectioning depth.

LMS provides a practical, *in situ* approach for visualizing and quantifying cell alignment, offering a broad spatial view of tissue organization. In contrast, direct assessment of PCP protein localization (e.g., VANGL2) requires optimized immunostaining and high-resolution imaging to resolve subcellular localization, making it technically more challenging. Although LMS is a powerful tool, several considerations must be taken into account for its application to Wnt/PCP studies.

First, LMS is an indirect readout of Wnt/PCP signaling, reflecting downstream morphology rather than direct molecular activity, so it may not capture all aspects of the pathway. Second, correct measurement of LMS to reflect the chondrocyte orientation requires longitudinal sections of limb tissues, and similar considerations would be necessary in other tissues with appropriate positive controls. Third, LMS requires case-by-case optimization of the neighborhood radius. In cartilage of E18.5 limbs, we used 30 *μ*m to ensure inclusion of 10 –20 neighbors, but this parameter may need adjustment depending on cell number and density within a given tissue or region. Fourth, because LMS does not account for orientation directionality, it cannot distinguish between clockwise and counterclockwise alignment, which could be important in contexts such as dorsal–ventral patterning in the limbs [5]. In such cases, GOA would be a more appropriate metric, with adjustments to reflect directional deviations from the tissue axis. Finally, LMS values in the tibia and fibula are slightly higher than in the femur, because these smaller bones contain a higher proportion of edge-located chondrocytes, which often display orientations that deviate from central populations, leading to elevated LMS values (Fig.2D and 3D). So, removal of edge-located cells could improve sensitivity in detecting more subtle phenotypic differences.

With these considerations, LMS represents a reliable and versatile approach for detecting Wnt/PCP-associated morphological changes. Because LMS values can be directly mapped onto tissue sections, this method enables spatially resolved identification of Wnt/PCP-related defects within specific anatomical regions or cell populations. Integrating LMS with other spatial assays, such as immunofluorescence, *in situ* hybridization, or spatial transcriptomics, can reveal how orientation defects correspond to changes in PCP protein distribution or transcriptional profiles.

### Limitations of the C83S mouse model

Due to the cleft palate phenotype in C83S mice, we were unable to maintain breeding colonies or generate additional generations. As a result, our experiments relied on the initial CRISPR/Cas9-edited embryos, which reduced the numbers of available embryos with the desired genotype. Another limitation of this model is that it may not be fully heterozygous due to incomplete efficiency of CRISPR/Cas9, or biallelic editing in some embryos due to too high efficiency. Although we selected embryos with approximately 50% correct template-mediated repair based on next-generation sequencing, we cannot exclude that some cells carry edits on both alleles. Despite its limitations, the current CRISPR-based C83S model remains valuable for studying C83S function, as heterozygous Wnt5a loss does not generate obvious phenotypes, and complete loss of Wnt5a produces a phenotype distinct from C83S mutants.

### Interpreting the mechanism of WNT5A-C83S in Robinow Syndrome

We conclude that the *Wnt5a-*C83S variant causes limb defects in mice through a mechanism that is neither loss-of-function nor dominant-negative, based on the following evidence:

- Wnt5a knockout models (both conditional and global) exhibit limb phenotypes only in homozygotes, not in heterozygotes, suggesting Wnt5a haploinsufficiency is generally well tolerated in mice. Thus, heterozygous *WNT5A* variants in human RS patients, such as C83S, would not have caused limb defects if they acted purely through a loss-of-function mechanism.
- To our knowledge, no nonsense or frameshift *WNT5A* variants that generate premature stop codons (i.e., null alleles) have been reported as dominant disease-causing, suggesting that a simple reduction in *WNT5A* expression is insufficient to cause diseases. In contrast, haploinsufficiency is well documented for other dominant disorders, such as NF1 for neurofibromatosis and PTEN for Cowden syndrome 1.
- In our study, *Wnt5a-*C83S mutation in mice did not cause location-specific chondrocyte orientation defects among limb elements, unlike the regionally patterned disruption seen in *Wnt5a-*cKO loss-of-function models (Fig.2D).
- *Wnt5a-*C83S did not reduce SOX9 expression in chondrocytes, in contrast to the *Wnt5a-*cKO model, which shows SOX9 downregulation (Fig.2D), consistent with findings in global Wnt5a knockout limbs [12, 14].
- *Wnt5a-*C83S did not cause severe digit abnormalities, which are hallmark features of Wnt5a loss-of-function limbs (Fig.2B and 3B).
- In our *in vitro* luciferase-based assays with both wild-type and C83S WNT5As in the same system, WNT5A-C83S did not exert dominant-negative effects on wild-type Wnt5a signaling (Fig.5).

Collectively, our *in vitro* and *in vivo* findings indicate that *WNT5A*-C83S functions as a hypomorphic variant with features consistent with gain-of-function, rather than exhibiting dominant-negative activity. However, in humans (not mice), possibly even modest reductions or alterations in WNT5A signaling dynamics may suffice to cause developmental abnormalities. The mutant protein could modify signaling outcomes in subtle, context-dependent ways that are difficult to detect *in vitro* or in mouse models. Therefore, while WNT5A-C83S does not directly antagonize wild-type WNT5A in our assays, it may nonetheless lead to dominant developmental phenotypes through a combination of haploinsufficiency and context-dependent signaling interference in human.

Finally, because RS is rare and obtaining sufficient patient-derived data for detailed phenotypic analysis remains challenging, phenotypic differences must be interpreted in the context of the causal gene (e.g., ligand versus receptor) and whether the mutation acts dominantly or recessively. Our findings therefore provide a critical step toward resolving the long-standing uncertainty regarding the mechanism of RS-associated mutations.

## Materials and Methods

### Mouse models

The mice used in this study were maintained in accordance with institutional animal care and use guidelines. Experimental protocols were approved by the Institutional Animal Care and Use Committee of the Van Andel Institute. Mice were fed LabDiet 5021 mouse breeder diet and housed in Thoren Maxi-Miser IVC caging systems with a 12-hour light/12-hour dark cycle. The *Wnt5a-*flox/flox mouse model was kindly provided by Dr. Terry P. Yamaguchi (National Cancer Institute, NIH) [34]. **Wnt5a-cKO** mice were generated by crossing *Wnt5a-*flox/flox animals with Prrx1-Cre (strain 005584 from The Jackson Laboratory) to conditionally delete Wnt5a in limb mesenchyme [19]. **Wnt5a-LSL** mice carry a loxP-STOP-loxP (LSL) cassette inserted upstream of the Wnt5a gene in the ROSA26 locus, allowing Prrx1-Cre-dependent ectopic expression of Wnt5a [14]. ***Wls-*cKO and *Lrp5/6-*cKO** models were generated by crossing Col2a1-Cre mice with *Wls-*flox or *Lrp5/6-*floxed mice to delete Wntless or canonical Wnt co-receptor Lrp5 and Lrp6 specifically in chondrocytes [35–37]. All embryos were harvested at E18.5 unless otherwise specified. ***Wnt5a*-C83S** *Wnt5a-*C83S knock-in mice harbor a CRISPR/Cas9-engineered point mutation (p.Cys83Ser) in the endogenous Wnt5a gene. The sgRNAs from IDT (GAAACTCTGCCACTTGTATC; crRNA and tracrRNA) were incubated with Cas9 protein (IDT) to form ribonucleoprotein (RNP) complexes for microinjection into mouse zygotes, with the repair template sequence being ATATATCATAGGTGCACAGCCTCTCTGCAGCCAACTGGCAGGACTTTCTCAAGGACAGAAGAAACTCTCCCATTTATACCAAGACCACATGCAGTACATTGGAGAAGGTGCGAAGACAGGCATCAAGGAATGCCAGTACCAGTTCCGGCATCGGAGATGGAAC. Due to perinatal lethality caused by cleft palate, only F0 embryos (the first generation after the genome editing) were used for analysis. The genomic DNA was genotyped by next-generation sequencing, in which the ~250 bp target region flanked by PCR primers with unique 8 bp barcodes was amplified, gel-verified, pooled, sequenced (Amplicon EZ, Genewiz/Azenta), aligned to the wild-type Wnt5a sequence using BWA-MEM, demultiplexed, and visualized in IGV to identify target modifications.

### Skeletal preparations

E18.5 embryonic skeletons were stained with alcian blue and alizarin red to visualize cartilage and bone, respectively. Briefly, embryos were eviscerated and fixed for 2 days in 95% ethanol. They were stained overnight at room temperature in 0.03% (w/v) Alcian Blue solution (Sigma-Aldrich, A3157) containing 80% ethanol and 20% glacial acetic acid. Samples were then destained in 95% ethanol for 24 hours, followed by pre-clearing in 1% KOH overnight at room temperature. Skeletons were subsequently stained overnight in 0.005% Alizarin Red solution (Sigma-Aldrich, A5533) containing 1% KOH. A second clearing step was performed by incubating tissues in 20% glycerol / 1% KOH solution for 24 hours. Finally, samples were transferred to 50% glycerol / 50% ethanol for imaging.

### Micro-Computed Tomography (micro-CT)

Limbs and skulls from E18.5 embryos were fixed in 10% neutral buffered formalin (NBF) at room temperature for 48 hours, followed by storage in 70% ethanol. Fore- and hindlimbs and skulls were scanned at an X-ray voltage of 50 kV, a current of 201 *μ*A, and with a 0.5 mm aluminum filter using the SkyScan 1172 Micro-CT system (Bruker Micro-CT: Kontich, Belgium). A pixel resolution of 2000 × 1200 and voxel size of 8 *μ*m were used. Images were reconstructed using NRecon 1.7.4.6. A volume of interest (VOI) (DataViewer 1.5.6.3) and region of interest (CTAn 1.18.8.0) were defined for each sample. A 3-dimensional rendered model was visualized in CTVol 2.2.1.0.

### Histology

Embryonic limb tissues were fixed overnight in 4% paraformaldehyde at 4°C, dehydrated, and embedded in paraffin. Longitudinal sections (5 μm) were cut through the hindlimbs for histological and immunofluorescent analysis. **Multiplex immunofluorescence (CycIF)** was performed on paraffin-embedded mouse limb sections using a protocol adapted from Eng et al [20]. Sections were deparaffinized in CitriSolv, rehydrated through graded ethanol, and rinsed in distilled water. For antigen retrieval, sections were treated with 2500 U hyaluronidase at 37°C for 1 hour, followed by heating in 10 mM citrate buffer (pH 6.0) using a microwave to sub-boiling temperature for 10 minutes and cooled to room temperature. Autofluorescence was quenched with freshly prepared 6% hydrogen peroxide in 250 mM NaHCO3 under light for 30 minutes. Images were acquired after quenching to document baseline background. The first staining round included Col2 (mouse, cytoplasm and membrane signal) and Sox9 (rabbit, nuclear signal) antibodies to maximize signal quality. Subsequent rounds included WGA (Biotium, 5 *μ*g/mL in PBS), ColX, and a monoclonal anti-muscle antibody. ColX was stained later due to its muscle-binding background, and the anti-muscle antibody was used last because of its strong signal. DAPI was included in each round for nuclear registration. After each imaging round, antibodies were removed by repeated H_2_O_2_ quenching and mild BME stripping at 56°C for 20 minutes. All cycles were registered with Warpy in QuPath, and background-corrected images were used for marker quantification. The antibodies are from Developmental Studies Hybridoma Bank (DSHB): SOX9 (Rabbit IgG, CPTC-SOX9–1, DSHB) 1:1000; skeletal muscle (Mouse IgG1, 12/101, DSHB) 1:200; COL2A1 (Mouse IgG1, II-II6B3, DSHB) 1:1000; COLX (Mouse IgG1, X-AC9, DSHB) 1:40; all primary antibodies were incubated overnight at 4 °C and secondary antibodies for 1 h at room temperature. Images were taken via Zeiss Axioscan Z1 scope at 20X magnification and saved in 16-bit czi files.

### Cell segmentation and classification

Briefly, cell segmentation and classification were performed in QuPath. Because chondrocytes and myocytes cluster together in limb tissue, these regions were sequentially selected using the annotation tool. As shown in supp_Fig.2, for Col2/ColX/muscle-positive regions, a square annotation was drawn around the positive area and refined using the SAM (Segment Anything Model, Meta) extension. Col2a1-positive chondrocytes were segmented with Cellpose using the Col2a1 channel, whereas other cell types were segmented from DAPI and WGA signals using InstanSeg model within QuPath. Myocyte segmentation can be prone to over splitting when using DAPI and WGA due to their multinucleated structure; importantly, these segmented myocytes preserved quantifiable orientation consistent with whole-muscle alignment. Hypertrophic chondrocytes were identified as chondrocytes (Col2a1-positive) located within ColX-positive regions/annotations, whereas myocytes were classified as cells within Muscle-positive regions/annotations. With this sequential classification approach, cells were classified as chondrocyte (Col2+), chondro-hyper (Col2+ and ColX+), myocyte (Muscle+), or other (negative for all). Finally, a line was manually drawn to separate the Col2+ region at the curved bone interface (see Fig.1D), with chondrocytes in the portion closer to the bone marrow re-classified as chondro-PZ. Nuclei were segmented with the StarDist model and paired with their corresponding cell objects by finding the nearest cell object using a kd-tree algorithm; nuclei not contained within any cell were excluded from analysis, likely due to weak WGA or Col2a1 signal. Compartmental measurements were then performed on matched nucleus–cell pairs. Cell objects segmented with InstanSeg from DAPI and WGA signals already contained both cell and nucleus compartments. For supplemental H&E-based segmentation using InstanSeg, small hematopoietic cells from bone marrow were manually excluded in QuPath and further filtered by cell size. Detailed segmentation procedures are provided in the shared scripts.

### Chondrocyte orientation quantification

Cell orientation was measured using two metrics. **Global orientation angle (GOA)** quantifies the absolute angle between each cell’s major axis (longest dimension) and the proximal–distal axis of the bone. In QuPath, the entire image was rotated separately for each annotation (containing segmented cell objects) so that the proximal–distal axis of the bone containing that annotation aligned vertically (the rotation angle was recorded in the annotation name). This rotation angle was then subtracted from the Feret angle of each segmented cell. The detections (cells) in each annotation were converted to ImageJ ROIs to calculate Feret angles. The angles were further normalized to an absolute 0–90° range, so that GOA values near 0° indicate alignment parallel to the proximal–distal axis, whereas values near 90° indicate perpendicular orientation. Although GOA does not directly measure alignment strength, its population distribution reflects it: a narrow distribution around a given angle indicates strong alignment, while a broad distribution indicates weak alignment. **Local misalignment score (LMS)** was calculated as the mean angular difference between a given cell and its neighbors within a 30 μm radius. As with GOA, cell orientation angles in all annotations were measured relative to a single proximal–distal axis (any consistent axis suffices).For each cell, the smallest possible difference between two orientations (accounting for 180° symmetry) was computed and remapped so that perfectly random orientations yield an LMS of 45°, constraining all values to the 0–45° range. Because cell orientation is periodic every 180°, the absolute angular difference between two randomly oriented cells is uniformly distributed between 0° and 90°, with an expected mean of 45°. This makes 45° the theoretical LMS maximum for a cell, corresponding to its neighborhood with completely random orientations.

### Cell culture

C3H10T1/2 cell line was purchased from ATCC and cultured in DMEM with 10% FBS and 1% penicillin–streptomycin at 37 °C, 5% CO_2_. The Wnt/PCP KIF26B reporter plasmid was generated by fusing the C-terminal region of mouse KIF26B (amino acids 1735–2112) to NanoLuc luciferase and cloning into a PiggyBac vector with a hygromycin resistance cassette (PB-(PGK-H2B-Citrine)/SV40-Hygro-SV40pA). C3H10T1/2 cells were co-transfected with this construct and a PiggyBac transposase helper plasmid using FuGENE X-tremeGENE^™^ (Sigma-Aldrich), then selected in hygromycin (400 *μ*g/mL) for 10–14 days before isolating clones with optimal responses to WNT5A treatments. Inducible expression constructs for Wnt5a variants or EGFP were generated in a PiggyBac tet-ON vector (PB-TA-ERP2, Addgene #80477) and introduced into C3H10T1/2-KIF26B/NanoLuc cells. Polyclonal pools of puromycin-resistant cells were expanded and validated for transgene expression and reporter activity using Nano-Glo luciferase assays (Promega). For Western blotting, cells were lysed in RIPA buffer supplemented with Roche cOmplete^™^ Mini Protease Inhibitor Cocktail (EDTA-free), and equal protein amounts were separated by SDS–PAGE and transferred to PVDF membranes. Membranes were probed with antibodies against V5 (CST 13202), DVL2 (CST 3206), DVL3 (CST 3218), phospho-VANGL2 (Invitrogen MA5–38242), and total VANGL2 (Millipore ABN2242), followed by HRP-conjugated secondary antibodies and ECL detection with a ChemiDoc MP imager (BioRad).

### WNT secretion assay

A NanoLuc–WNT5A plasmid (pHAGE2-Blast-NanoLuc-hWNT5A; gift from Dr. Adrian Salic, Harvard Medical School) was used to generate the C83S variant (c.248G>C; p.Cys83Ser) via site-directed mutagenesis (Q5 kit, NEB), and the mutation was confirmed by Sanger sequencing (Genewiz/Azenta). As described in the original publication [38], HEK293T cells were transiently transfected with NanoLuc-tagged WNT5A (WT or C83S) or NanoLuc-tagged WNT3A plasmids using FuGENE HD (Promega). For secretion assays, fresh media was added to transfected cells, and conditioned media were collected at indicated time points after media replacement, clarified by centrifugation, and NanoLuc activity in the supernatant was measured using the Nano-Glo Luciferase Assay (Promega). Extracellular NanoLuc signal was normalized to total NanoLuc measured from corresponding cell lysates harvested at the end of the assay. Where indicated, cells were treated with the PORCN inhibitor LGK974 (2 *μ*M) to confirm PORCN dependence.

### Statistical analysis

Orientation metrics (GOA and LMS) were analyzed using linear mixed-effects models (nlme package, R). Models included genotype, location (or cell type), and their interaction as fixed effects, with a random intercept for embryo to account for biological replicates. Variance heterogeneity across locations was allowed when supported by the data. Median values per embryo per location were used as the dependent variable, as individual measures did not follow standard parametric distributions. Model assumptions were checked using regression diagnostics and were adequately met. Global orientation angle (GOA) distributions were compared using a non-parametric permutation test based on the Wasserstein distance (transport package, R). The test statistic was defined as the ratio of average between-group to within-group Wasserstein distances, calculated using Gini’s mean difference. This framework is analogous to an F statistic but non-parametric and more sensitive to differences in both scale and dispersion.

## Supplementary Material

1

## Data Availability

All image analysis and quantification scripts will be made available at the time of publication via a public GitHub repository. Raw image data and segmentation results are available upon request.

## Key Findings and Study Highlights

- We established a novel image-based method, the local misalignment score (LMS), to measure chondrocyte orientation in long bones, independent of embryo age or sectioning depth. LMS provides a reliable *in vivo* morphological readout of defective Wnt/PCP-associated phenotypes and enables visualization and quantification of alignment in chondrocytes as well as other cell types within and beyond cartilage tissues.
- Wnt5a gradient-dependent limb phenotypes: Alterations in Wnt5a expression, whether reduced or elevated, produced limb defects with region-specific severity that correlated with deviations from the endogenous Wnt5a expression gradient.
- *Wnt5a-*C83S mouse model: Using CRISPR/Cas9 mutagenesis, we generated a mouse model with a heterozygous C83S mutation, mimicking the heterozygous state in human RS patients. Unlike the spatially patterned severity seen in Wnt5a knockout or ectopic expression models, the C83S model shows a uniformly severe phenotype across limb regions.
- We adapted a new and robust luciferase-based reporter system (KIF26B-NanoLuc) in C3H10T1/2 cells to measure Wnt5a activity *in vitro*, and showed that wild-type WNT5A, but not the C83S variant, activated the readouts. Contrary to expectations of a dominant-negative effect, the C83S variant did not inhibit wild-type Wnt5a signaling in cell culture models of autocrine, juxtracrine, or paracrine activity.

Together with genetic evidence from Robinow Syndrome (RS) patients, our findings suggest that the C83S variant contributes to RS through a putative gain-of-function mechanism rather than loss-of-function or dominant-negative effects, providing new insights into the pathogenic mechanisms of RS and the complex regulation of Wnt/PCP signaling during limb development.

## References

[1] Zhang C., , Novel pathogenic genomic variants leading to autosomal dominant and recessive Robinow syndrome. Am J Med Genet A, 2021. 185(12): p. 3593–3600. 33048444 10.1002/ajmg.a.61908 PMC8445516

[2] Butler M.T. and Wallingford J.B., Planar cell polarity in development and disease. Nat Rev Mol Cell Biol, 2017. 18(6): p. 375–388. 28293032 10.1038/nrm.2017.11 PMC5826606

[3] Yang Y. and Mlodzik M., Wnt-Frizzled/planar cell polarity signaling: cellular orientation by facing the wind (Wnt). Annu Rev Cell Dev Biol, 2015. 31: p. 623–46. 26566118 10.1146/annurev-cellbio-100814-125315 PMC4673888

[4] White J.J., , WNT Signaling Perturbations Underlie the Genetic Heterogeneity of Robinow Syndrome. Am J Hum Genet, 2018. 102(1): p. 27–43. 29276006 10.1016/j.ajhg.2017.10.002 PMC5777383

[5] Gao B. and Yang Y., Planar cell polarity in vertebrate limb morphogenesis. Curr Opin Genet Dev, 2013. 23(4): p. 438–44. 23747034 10.1016/j.gde.2013.05.003 PMC3759593

[6] Pate M., , IS1245 RFLP-based genotyping study of Mycobacterium avium subsp. hominissuis isolates from pigs and humans. Comp Immunol Microbiol Infect Dis, 2008. 31(6): p. 537–50. 18243315 10.1016/j.cimid.2007.10.002

[7] Kunkel S.L., Through the looking glass: the diverse in vivo activities of chemokines. J Clin Invest, 1999. 104(10): p. 1333–4. 10562292 10.1172/JCI8511 PMC409848

[8] Dictenberg J.B., , A direct role for FMRP in activity-dependent dendritic mRNA transport links filopodial-spine morphogenesis to fragile X syndrome. Dev Cell, 2008. 14(6): p. 926–39. 18539120 10.1016/j.devcel.2008.04.003 PMC2453222

[9] Person A.D., , WNT5A mutations in patients with autosomal dominant Robinow syndrome. Dev Dyn, 2010. 239(1): p. 327–37. 19918918 10.1002/dvdy.22156 PMC4059519

[10] Gignac S.J., , Robinow syndrome skeletal phenotypes caused by the WNT5AC83S variant are due to dominant interference with chondrogenesis. Hum Mol Genet, 2019. 28(14): p. 2395–2414. 31032853 10.1093/hmg/ddz071 PMC6606851

[11] Hosseini-Farahabadi S., , Abnormal WNT5A Signaling Causes Mandibular Hypoplasia in Robinow Syndrome. J Dent Res, 2017. 96(11): p. 1265–1272. 28662348 10.1177/0022034517716916

[12] Yamaguchi T.P., , A Wnt5a pathway underlies outgrowth of multiple structures in the vertebrate embryo. Development, 1999. 126(6): p. 1211–23. 10021340 10.1242/dev.126.6.1211

[13] Tai C.C., , Wnt5a knock-out mouse as a new model of anorectal malformation. J Surg Res, 2009. 156(2): p. 278–82. 19577771 10.1016/j.jss.2009.03.087 PMC3412158

[14] Gao B., , Coordinated directional outgrowth and pattern formation by integration of Wnt5a and Fgf signaling in planar cell polarity. Development, 2018. 145(8). 10.1242/dev.163824 PMC5964654 29615464

[15] Yang Y., , Wnt5a and Wnt5b exhibit distinct activities in coordinating chondrocyte proliferation and diWerentiation. Development, 2003. 130(5): p. 1003–15. 12538525 10.1242/dev.00324

[16] Gros J., , WNT5A/JNK and FGF/MAPK pathways regulate the cellular events shaping the vertebrate limb bud. Curr Biol, 2010. 20(22): p. 1993–2002. 21055947 10.1016/j.cub.2010.09.063 PMC2998074

[17] Farrera-Hernandez A., Marin-Llera J.C., and Chimal-Monroy J., WNT5A-Ca(2+)-CaN-NFAT signalling plays a permissive role during cartilage diWerentiation in embryonic chick digit development. Dev Biol, 2021. 469: p. 86–95. 33058830 10.1016/j.ydbio.2020.10.003

[18] Navajas Acedo J., , PCP and Wnt pathway components act in parallel during zebrafish mechanosensory hair cell orientation. Nat Commun, 2019. 10(1): p. 3993. 31488837 10.1038/s41467-019-12005-y PMC6728366

[19] Logan M., , Expression of Cre Recombinase in the developing mouse limb bud driven by a Prxl enhancer. Genesis, 2002. 33(2): p. 77–80. 12112875 10.1002/gene.10092

[20] Eng J., , A framework for multiplex imaging optimization and reproducible analysis. Commun Biol, 2022. 5(1): p. 438. 35545666 10.1038/s42003-022-03368-y PMC9095647

[21] Zhou X., , Chondrocytes transdiWerentiate into osteoblasts in endochondral bone during development, postnatal growth and fracture healing in mice. PLoS Genet, 2014. 10(12): p. e1004820. 25474590 10.1371/journal.pgen.1004820 PMC4256265

[22] Joeng K.S., , Lrp5 and Lrp6 redundantly control skeletal development in the mouse embryo. Dev Biol, 2011. 359(2): p. 222–9. 21924256 10.1016/j.ydbio.2011.08.020 PMC3220949

[23] Holmen S.L., , Essential role of beta-catenin in postnatal bone acquisition. J Biol Chem, 2005. 280(22): p. 21162–8. 15802266 10.1074/jbc.M501900200

[24] Joiner D.M., , LRP5 and LRP6 in development and disease. Trends Endocrinol Metab, 2013. 24(1): p. 31–9. 23245947 10.1016/j.tem.2012.10.003 PMC3592934

[25] Shulman H.J. and Meyer K., Protein-polyaccharide of chicken cartilage and chondrocyte cell cultures. Biochem J, 1970. 120(4): p. 689–97. 4250334 10.1042/bj1200689 PMC1179661

[26] Zhang B., , A human embryonic limb cell atlas resolved in space and time. Nature, 2024. 635(8039): p. 668–678. 38057666 10.1038/s41586-023-06806-x PMC7616500

[27] Chen J., , WNT7B promotes bone formation in part through mTORC1. PLoS Genet, 2014. 10(1): p. e1004145. 24497849 10.1371/journal.pgen.1004145 PMC3907335

[28] Bi W., , Sox9 is required for cartilage formation. Nat Genet, 1999. 22(1): p. 85–9. 10319868 10.1038/8792

[29] Kozhemyakina E., Lassar A.B., and Zelzer E., A pathway to bone: signaling molecules and transcription factors involved in chondrocyte development and maturation. Development, 2015. 142(5): p. 817–31. 25715393 10.1242/dev.105536 PMC4352987

[30] Bradley E.W. and Drissi M.H., WNT5A regulates chondrocyte diWerentiation through diWerential use of the CaN/NFAT and IKK/NF-kappaB pathways. Mol Endocrinol, 2010. 24(8): p. 1581–93. 20573686 10.1210/me.2010-0037 PMC5417459

[31] Susman M.W., , Kinesin superfamily protein Kif26b links Wnt5a-Ror signaling to the control of cell and tissue behaviors in vertebrates. Elife, 2017. 6. 10.7554/eLife.26509 PMC5590807 28885975

[32] Karuna E.P., , Identification of a WNT5A-Responsive Degradation Domain in the Kinesin Superfamily Protein KIF26B. Genes (Basel), 2018. 9(4). 10.3390/genes9040196 PMC5924538 29621187

[33] Willert K., , Wnt proteins are lipid-modified and can act as stem cell growth factors. Nature, 2003. 423(6938): p. 448–52. 12717451 10.1038/nature01611

[34] Miyoshi H., , Wnt5a potentiates TGF-beta signaling to promote colonic crypt regeneration after tissue injury. Science, 2012. 338(6103): p. 108–13. 22956684 10.1126/science.1223821 PMC3706630

[35] Carpenter A.C., , Generation of mice with a conditional null allele for Wntless. Genesis, 2010. 48(9): p. 554–8. 20614471 10.1002/dvg.20651 PMC3689319

[36] Holmen S.L., , Decreased BMD and limb deformities in mice carrying mutations in both Lrp5 and Lrp6. J Bone Miner Res, 2004. 19(12): p. 2033–40. 15537447 10.1359/JBMR.040907

[37] Ovchinnikov D.A., , Col2a1-directed expression of Cre recombinase in diWerentiating chondrocytes in transgenic mice. Genesis, 2000. 26(2): p. 145–6. 10686612

[38] de Almeida Magalhaes T., , Extracellular carriers control lipid-dependent secretion, delivery, and activity of WNT morphogens. Dev Cell, 2024. 59(2): p. 244–261 e6. 38154460 10.1016/j.devcel.2023.11.027 PMC10872876

