# Supplementary material for: Local Misalignment Scoring Reveals Spatially Uniform Chondrocyte Disorganization in a Wnt5a-C83S Knock-in Model of Robinow Syndrome
Source: Res Sq. 2025 Dec 17:rs.3.rs-8283811. Preprint. [Version 1] doi: 10.21203/rs.3.rs-8283811/v1 (PMC12776500; doi:10.21203/rs.3.rs-8283811/v1)
Supplement: 1 [file NIHPPRS8283811V1-supplement-1.pdf]

Supplemental

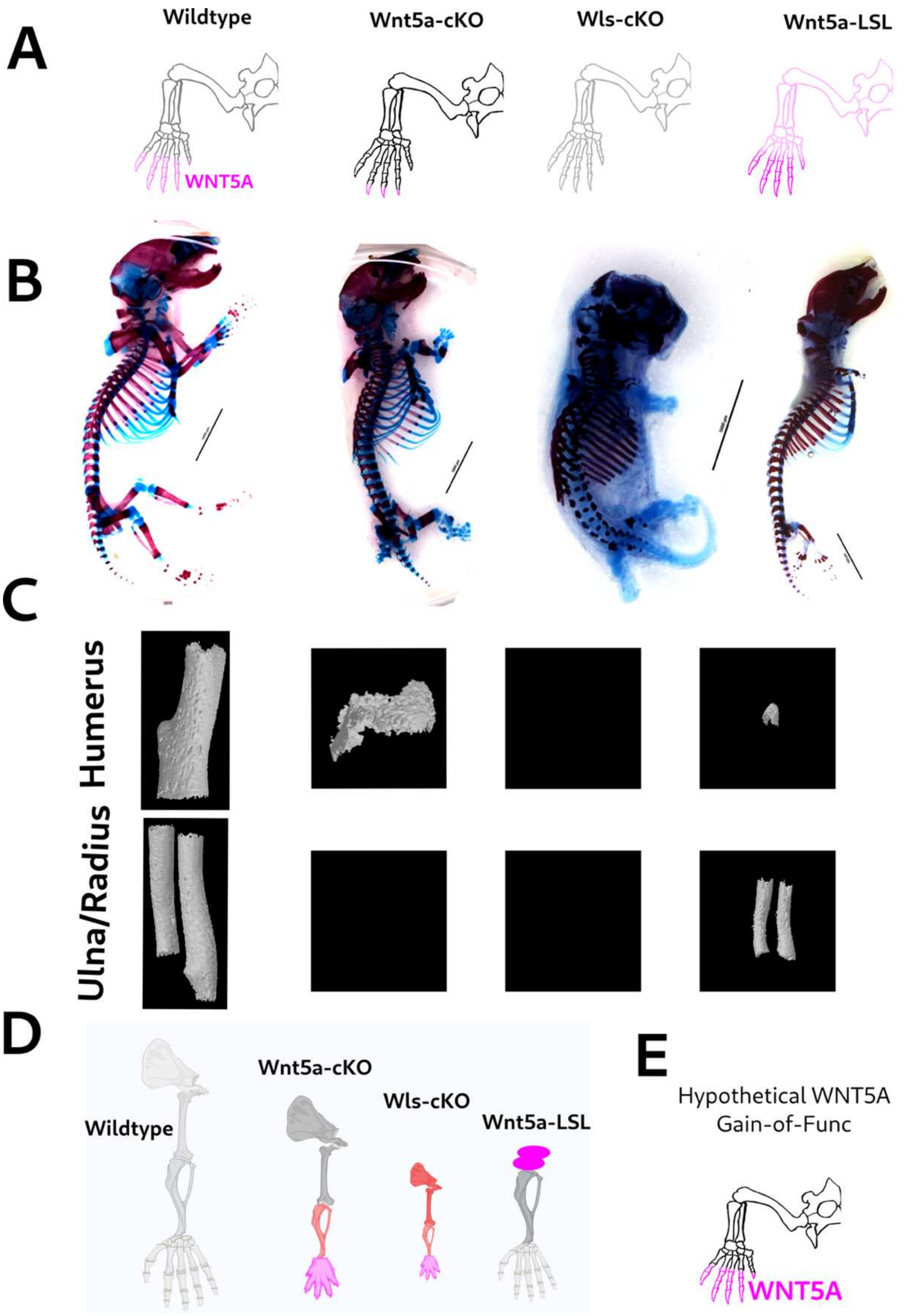

Suppl_Fig. 1 Limb-specific conditional mouse models and their phenotypes

- A. Diagram of Prrx1-Cre–driven conditional mouse models. The hind limb diagram illustrates Wnt expression patterns: endogenous Wnt5a in magenta and other Wnt ligands in black. In *Wnt5a*-LSL, Wnt5a is ectopically expressed throughout the limb bud at lower-than-endogenous levels (driven by the ROSA26 promoter in the LSL cassette). In *Wls*-cKO, secretion of all Wnt ligands is blocked in the limb bud, with gray indicating absence of Wnt secretion, resulting in severe limb phenotypes.
- B. Whole-mount skeletal staining of E18.5 mouse embryos. Red: mineralized bone; Blue: cartilage. Note, *Wls*-cKO shows shortened facial elements due to the Prrx1-Cre activity in the craniofacial mesenchyme, while *Wnt5a*-cKO alone does not show a severe facial phenotype, suggesting other Wnt ligands contribute to craniofacial development.
- C. Representative microCT reconstructions of E18.5 forelimb elements from the indicated mouse models. Mineralized bone elements are visualized in 3D to assess the extent of limb shortening and patterning defects.
- D. Schematic of limb elements showing phenotype severity based on microCT data. Red indicates severe shortening; magenta indicates extreme shortening with poor mineralization.
- E. Diagram of a hypothetical true Wnt5a gain-of-function limb bud, where elevated Wnt5a (magenta) occurs within the normal expression domain without disrupting the endogenous gradient.

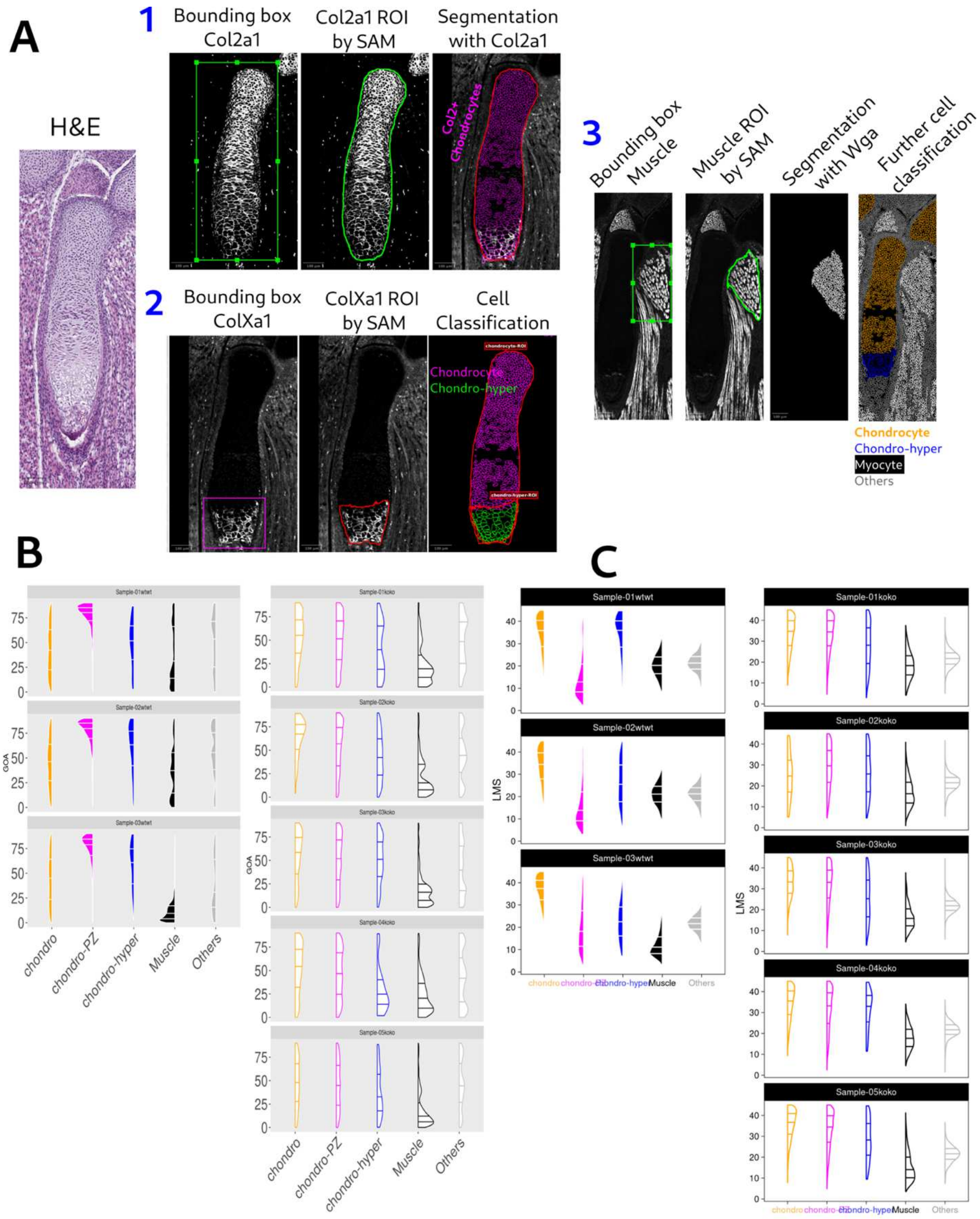

Suppl_Fig. 2 Cell-type classification in QuPath and embryo-level GOA/LMS analysis

- A. The H&E image shows the overall tissue morphology. (1) chondrocyte segmentation and classification: First, a bounding box is drawn around the region of Col2a1-positive cartilage. Second,

the region is further processed with Segment Anything Model (SAM) from Meta AI to obtain precise Col2a1-positive cartilage region. Third, the SAM output is used as the annotation for cellpose-based cell segmentation. The segmented cells are then classified as chondrocytes. (2) hypertrophic chondrocyte (chondrocyte-hyper) classification: SAM is used based on the ColXa1-positive hypertrophic chondrocyte region to draw an annotation of chondrocyte-hyper, within which all the chondrocytes are re-classified as chondrocyte-hyper. (3) myocyte segmentation and classification: Similar to chondrocyte segmentation, a bounding box is drawn around the region of Muscle-positive myocytes, which is further processed with SAM to obtain precise Muscle-positive myocyte region. Then myocytes are segmented using cellpose with Wga signal. These cells are classified as myocytes, and all other non-chondrocyte and non-myocyte cells are classified "Others" cell type. The specific codes for the cell segmentation and classification are available in the GitHub repository.

- B and C. The half violin plots show the distribution of GOA and LMS values of limb cells from each embryo. Each subplot represents one embryo, with the genotype and embryo ID indicated at the top. The cell types are indicated by color as well as labels on the bottom.

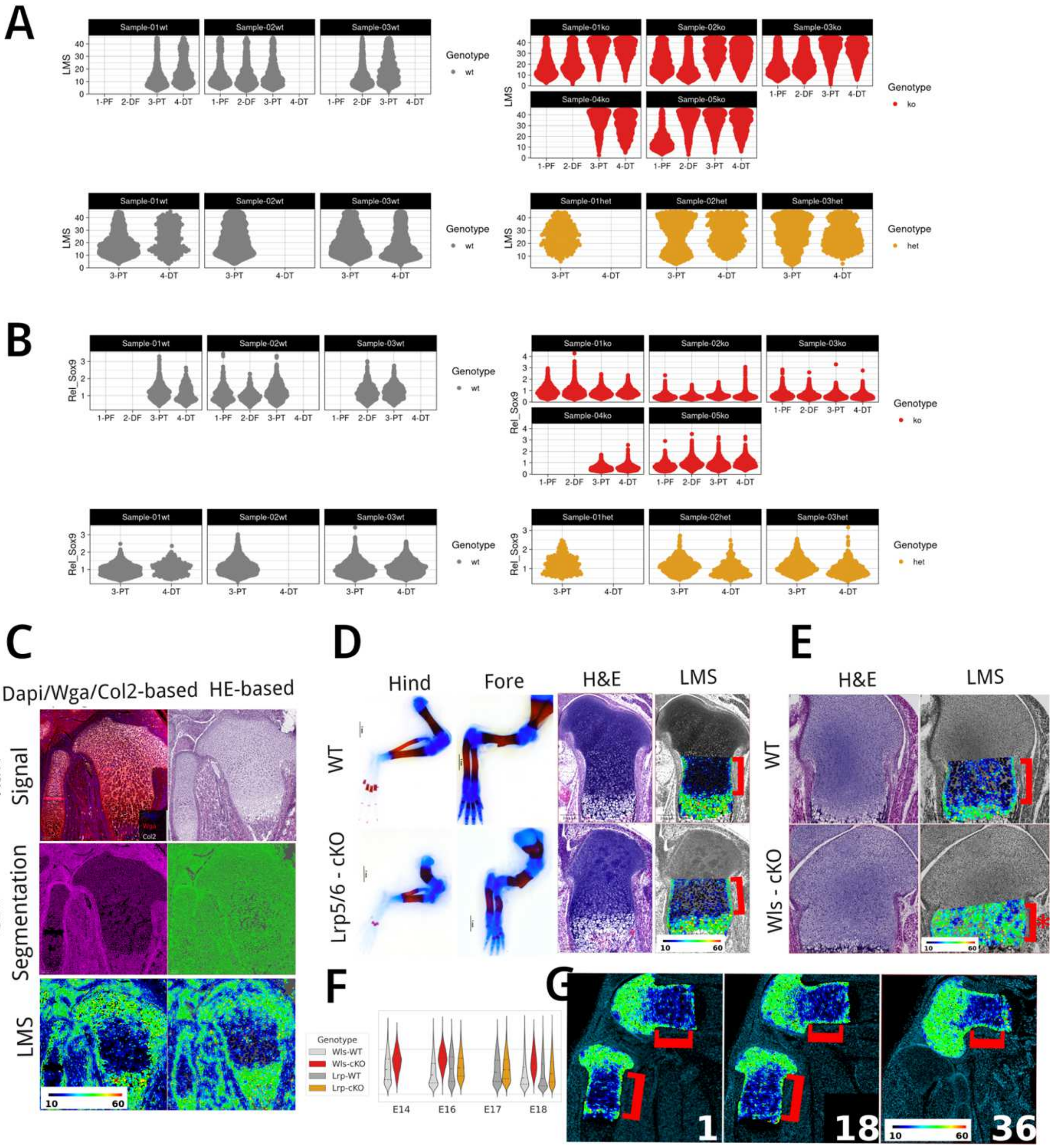

Suppl_Fig.3 Embryo-level LMS/SOX9 analysis and LMS independence from canonical Wnt signaling, embryo stage, sectioning depth, and measurement method

- A and B. LMS and SOX9 violin plots of chondro-PZ from wild-type and mutants from E18.5 embryos of *Wnt5a*-cKO and *Wnt5a*-LSL models. Each subplot represents one embryo, with the genotype and embryo ID indicated at the top. The bone element (1-proximal femur; 2-distal femur; 3-proximal tibia; 4-distal tibia) is indicated at the bottom. Note that not all bone elements are present in every embryo due to variability in sectioning planes.
- C. The cell segmentation and LMS analysis were performed on the same tissue as shown in Suppl_Fig2A using either Col2a1/Wga signal or H&E staining. The cell segmentation using Col2a1/Wga was performed as described insuppl_Fig. 2. The H&E staining was performed on the

same tissue section and segmented using the InstanSeg plugin in QuPath. Refer to Methods for details on segmentation with H&E staining.

- D. Violin plots of LMS values in chondrocytes from wild-type, *Wls*-cKO (Col2a1-Cre-driven), and *Lrp5/6*-cKO (Col2a1-Cre-driven) hind limbs across developmental stages (E14.5-E18.5). Note, the LMS values of hypertrophic chondrocytes are also included in the violin plots.
- E. Whole-mount skeletal staining of *Lrp5/6*-cKO limbs at E18.5. The *Lrp5/6*-cKO limbs are significantly shorter than wild-type limbs, but the orientation of chondrocytes in the growth plate is not affected.The heatmap of LMS of chondrocytes is overlaid on the gray H&E images. The approximate regions of chondro-PZ are indicated with red brackets.
- F. H&E and LMS heatmap of wildtype and *Wls*-cKO hind limbs at E18.5. The Wls cKO limbs are significantly shorter than wild-type limbs, and the orientation of chondrocytes in the growth plate is disrupted. The region of chondro-PZ are indicated with red brackets and the red star indicates a significant difference in LMS compared to wild-type control.
- G. Heatmaps depicting LMS of all chondrocytes in wild-type limbs across different sectioning depths. Section numbers are indicated on each image, with higher numbers corresponding to deeper tissue cuts (~180 Œºm depth in total). DAPI staining was used to visualize all cells within each section. Note that the tibia is nearly absent in the final (36) section

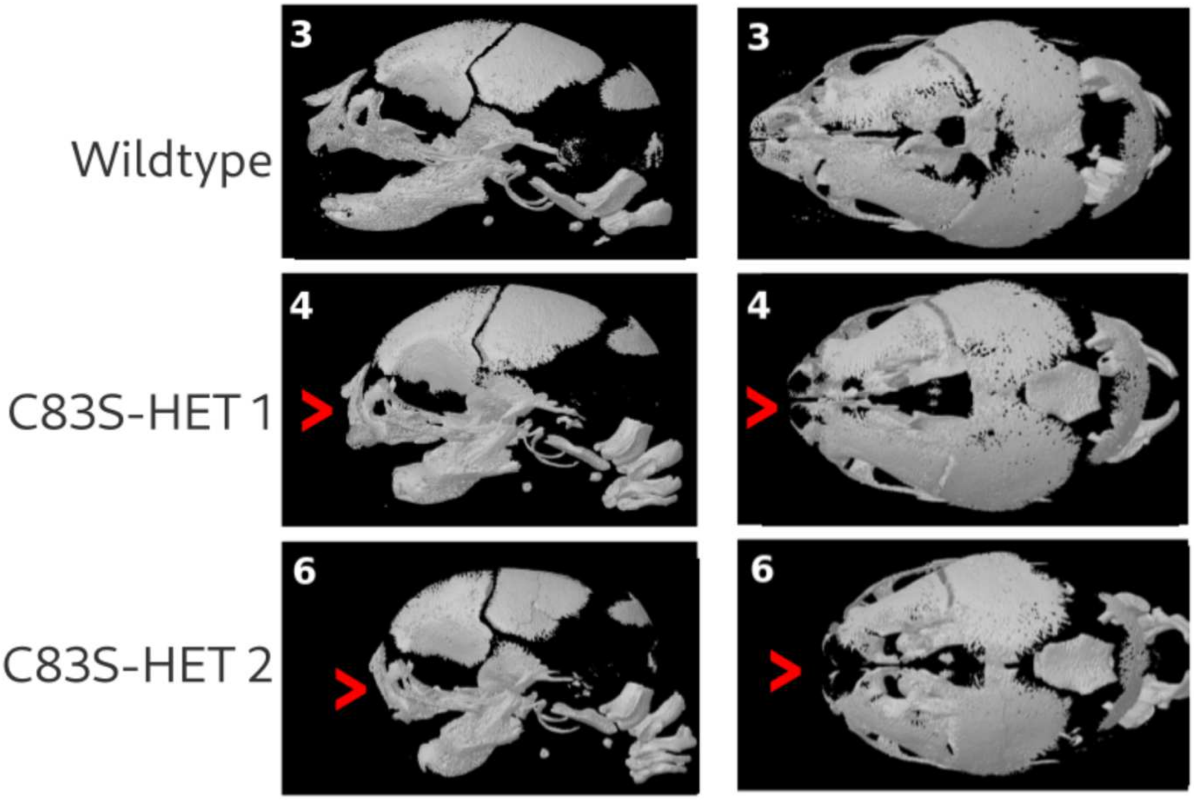

Suppl_Fig.4 MicroCT images of C83S skulls

MicroCT was performed on E18.5 wild-type and C83S skulls to assess craniofacial phenotypes. The images show 3D reconstructions of the skulls, with mineralized bone elements visualized in gray. The C83S skulls display shortened nasal regions (arrows) when viewed from the front and side.
